# A single-centre, randomised controlled feasibility pilot trial comparing performance of direct laryngoscopy versus videolaryngoscopy for endotracheal intubation in surgical patients

**DOI:** 10.1186/s40814-019-0433-6

**Published:** 2019-03-28

**Authors:** Alice Loughnan, Carolyn Deng, Felicity Dominick, Lora Pencheva, Doug Campbell

**Affiliations:** 1 Anaesthetic Department, Kings College Hospital, Ground floor Cheyne Wing, Denmark Hill, Brixton, London, SE5 9RS UK; 2 Auckland City Hospital, Auckland, New Zealand

**Keywords:** Endotracheal intubation, Direct laryngoscopy, Videolaryngoscopy, Airway management

## Abstract

**Introduction:**

Most trials comparing effectiveness of laryngoscopy technique use surrogate endpoints. Intubation success is a more appropriate endpoint for comparing effectiveness of techniques or devices. A large pragmatic clinical trial powered for intubation success has not yet been performed.

**Methods:**

We tested the feasibility of a randomised controlled trial to compare the performance of direct laryngoscopy versus videolaryngoscopy for endotracheal intubation. The trial was conducted in the Department of Adult and Emergency Anaesthesia at the Auckland City Hospital, New Zealand. Patients over 18 years who required endotracheal intubation and were not known or predicted to be difficult to bag-mask ventilate were eligible for the study. Patients were excluded if they required rapid sequence induction, fibreoptic intubation or were unable to consent due to language barriers or cognitive impairment.

Patients were permuted block randomised in groups of 8 to either direct laryngoscopy (DL) or videolaryngoscopy (VL) for the technique of endotracheal intubation. Patients were blinded to laryngoscopic technique; the duty anaesthetist, outcome assessors and statistician were unblinded.

Feasibility was assessed on recruitment rate, adherence to group assignment and data completeness. Primary outcome was first-pass success rate, with secondary outcomes of time to intubation (seconds), Intubation Difficulty Score and complication rate.

**Results:**

One hundred and six patients were randomised and 100 patient results were analysed. Completed data from patients randomised to the DL group (*n* = 49) was compared with those in the VL group (*n* = 51). Group adherence and data completeness were 100% and 97%, respectively. First-pass success rate was 83.7% in the direct laryngoscopy group and 72.5% in the videolaryngoscopy group (*p* = 0.18). Median time to intubation was significantly shorter for direct laryngoscopy when compared to videolaryngoscopy (34 s v 43 s, *p* = 0.038). Complications included mucosal trauma and airway bleeding which are recognised complications of endotracheal intubation.

**Conclusion:**

A large, pragmatic, multicentre, randomised controlled trial comparing the relative effectiveness of direct laryngoscopy and indirect videolaryngoscopy is feasible.

**Trial registration:**

Australian New Zealand Clinical Trials Registry (ANZCTR), ACTRN12615001267549

## Introduction

Most trials comparing effectiveness of laryngoscopy technique use surrogate endpoints such as laryngoscopic view or Intubation Difficulty Score. Intubation success is a more appropriate endpoint for comparing effectiveness of techniques or devices. Previous trials have small sample sizes, of between 50 and 200 participants, [1–3], and do not have an adequate sample size to show a clinically meaningful difference. In trials recording failed intubation, there is much heterogeneity in this definition, which makes drawing clear conclusions difficult [4]. These trials have focused on specific populations, making results less generalisable [5–7]. Using the laryngoscopic view as a surrogate measure for successful intubation can give false assurances of device efficacy [8, 9]. The correlation between view and intubation success is especially poor in indirect videolaryngoscopic techniques. The first attempt to intubate is often the most successful [10]. We believe the most meaningful endpoint to determine the performance of these devices is first-pass intubation success. Small improvements in first-pass success could translate to improved overall success curtailing the need for multiple attempts. Improved first-pass success should confer less airway trauma and possibly prevent deterioration to cannot intubate-cannot ventilate scenarios [11]. The Difficult Airway Society has highlighted the importance of a robust and high standard of evidence to assess the use of new devices in airway management [12]. Meta-analyses comparing these devices have amalgamated small trials using surrogate primary endpoints and have rarely collected data relating to harm that could be consequent to their use [13–15]. Evidence relating to different videolaryngoscopic techniques, such as direct versus indirect videolaryngoscopy, also require further examination, as devices with different designs are unlikely to perform equally [4].

The aim of the study was to assess the feasibility of a larger, multicentre randomised controlled trial. Feasibility outcomes were recruitment rate, acceptability of the trial protocol, eligibility criteria, and data completeness. First-pass intubation success rate was assessed to confirm the sample size calculation for a large, definitive randomised controlled trial.

## Methods

### Trial design

Ethical approval for this study (Ethical Committee ref. 15/CEN/199) was provided by the Health and Disability Ethics Committee, Wellington, New Zealand. A randomised controlled feasibility trial was conducted in the Department of Adult and Emergency Anaesthesia at Auckland City Hospital, New Zealand. The trial was designed to assess the feasibility of undertaking a larger pragmatic effectiveness trial examining the success rate of a videolaryngoscopy (VL) technique when compared to direct laryngoscopy (DL).

### Participants

Patients were enrolled between March 2016 and June 2017. Operating lists were screened the day before surgery by investigators for suitable patients and the duty anaesthetist assigned to the list was approached to participate in the trial. Suitable patients were assessed for eligibility and written informed consent obtained.

Patients over 18 years who required endotracheal intubation, where there was no contraindication to DL or VL, were included. Patients were excluded if they required an awake approach, rapid sequence induction or flexible bronchoscopic intubation; if they were a known difficult intubation; or if there were language or cognitive barriers that precluded adequate informed consent. Patients were also excluded if the operating anaesthetist did not have enough clinical experience in the use of DL or VL without in-theatre supervision. We defined adequate experience as having used a videolaryngoscope greater than 20 times in clinical practice. Patient consent was obtained by study investigators on the day of surgery in the form of a detailed discussion and patient information leaflets.

### Intervention

Patients were randomised to initial intubation attempt using either VL or DL. Laryngoscopy was performed by the duty anaesthetist assigned to that operating room. Within the VL group, the choice of device was selected by the duty anaesthetist. The GlideScope® AVL System (Verathon, Washington) and McGRATH-MAC™ (Medtronic, Minneapolis) are the most readily available videolaryngoscopes in the Department of Adult and Emergency Anaesthesia, Auckland City Hospital. A standard Macintosh blade was used in the group assigned to DL. Blade size, drugs, positioning and anaesthetic technique were at the discretion of the duty anaesthetist.

Patient demographics, airway examination findings, relevant comorbidities, intubation details and the duty anaesthetist’s grade and years of experience were also recorded. Airway examination findings were quantified using the Airway Difficulty Score (ADS) [16] (Table 1).

**Table 1:** Airway Difficulty Score (ADS). Used to quantify patient characteristics of difficult intubation [16]

	Score = 1	Score = 2	Score = 3
Thyromental distance (cm)	> 6	5–6	< 5
Mallampati Score	I	II	III & IV
Mouth opening (cm)	> 4	2–3	< 1
Neck mobility	Normal	Reduced	Fixed flexion
Upper incisors	Absent	Normal	Prominent

### Outcomes

Feasibility was assessed on recruitment rate, adherence to group assignment, data completeness and local incidence of first-pass intubation success. Successful pilot targets were defined as recruitment of 100 trial participants over a 12-month period, 95% data completeness, 95% adherence to group allocation and local incidence of first-pass success of less than or equal to 85%. A local incidence of first-pass intubation success greater than 85% would indicate a definitive trial would not be feasible as large recruitment numbers would be required to demonstrate a clinically relevant difference.

The primary endpoint was the rate of successful first-pass endotracheal intubation when comparing DL to VL. Successful first-pass intubation was defined as one fluid movement from the endotracheal tube entering the mouth to being positioned in the trachea, during a single apnoeic episode. Airway adjuncts or assistance such as bougie, stylet, optimal external laryngeal manipulation (OELM) or suctioning could be used prior to the endotracheal tube entering the patient’s oropharynx. Curtailing the attempt for bag-mask ventilation, repositioning, assistance or additional equipment to those already specified was deemed a failure.

Secondary endpoints were time to endotracheal intubation, Intubation Difficulty Score (IDS) [17] (Table 2) and complications. Time to intubation was defined as the time from laryngoscope in hand to when endotracheal intubation is confirmed by end-tidal capnography. Complications were assessed as a composite of airway bleeding, mucosal injury or dental damage in the Post Anaesthetic Care Unit (PACU).

**Table 2:** Intubation Difficulty Score (IDS). Used to quantify difficulty of intubation [17]

IDS parameter	Score
Number of attempts > 1	N1
Number of operators > 1	N2
Number of alternative techniques	N3
Cormack and Lehane grade minus 1	N4
Total IDS = sum of scores	

### Sample size

A total of 100 patients were estimated to be an adequate representation of our target population for the purposes of a pilot feasibility trial. Although formal sample size calculations are not required for pilot studies, we used a one sided 80% confidence interval approach based on our feasibility objectives [18, 19]. It is based upon a sample size sufficient to make a reliable estimate of the primary endpoint. We chose a ± 5% for the estimate of proportion, which was arbitrary, but was used to allow sufficiently accurate estimate of trial outcomes to make decisions for design of a larger trial.

### Randomisation

Patients were permuted blocked randomised in groups of 8 to either DL or VL. Randomisation was performed by a research coordinator who disclosed group assignation to the investigator. Details were contained in sealed opaque envelopes until enrolment into the study was completed.

### Blinding

Patients were blinded to laryngoscopic technique, but it was not possible to blind the duty anaesthetist. Outcome assessment was by one of four unblinded investigators present at time of intubation. All outcome assessors went through a standardised training process prior to trial initiation. Primary endpoint definition was well defined and instruction as to how to adjudicate was provided.

### Statistical analysis

Responses were collected and collated using Microsoft Excel (2017). Simple descriptive statistics were used for feasibility outcomes and baseline demographics. Continuous data such as time to intubate were tested for normality using histograms, probability-probability plots, skewness and kurtosis. Normally distributed data were summarised using mean and standard deviation, skewed data were summarised using median and interquartile range (Q1–Q3) and categorical variables were summarised using number and percent.

Categorical outcome variables such as first-pass success and complications were tested using Pearson’s chi-squared test. Continuous or ordinal variables such as time to intubate and IDS were tested using Wilcoxon rank-sum (Mann-Whitney) tests. 95% confidence intervals were reported for outcome measures. A *p* value of < 0.05 was deemed statistically significant. All statistical analyses were conducted using Stata/IC (StataCorp. 2017. Statistical Software: Release 15.0. College Station, TX: StataCorp LLC).

## Results

**Fig. 1:**
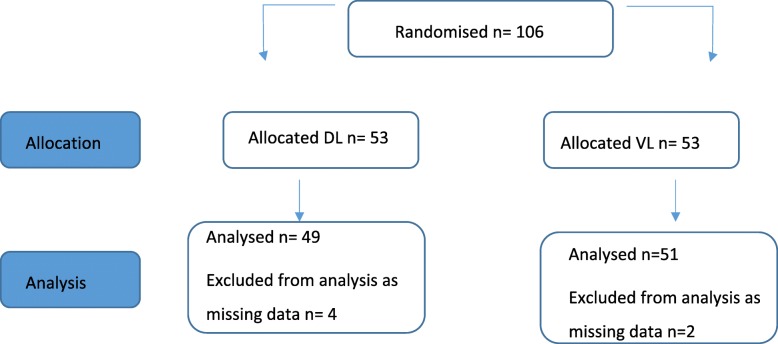
Flowchart with patient randomisation, group allocation and analysis. *n* = number of patients per group

Records of patients screened for eligibility, excluded or declined consent were not recorded. Patient enrolment started in January 2016 and finished in June 2017; 106 patients were randomised and 100 patient results were analysed. Six patient’s results were incomplete and therefore not included in analysis. Completed data from patients randomised to the DL group (*n* = 49) was compared with those in the VL group (*n* = 51), see flowchart in Fig. 1.

Baseline demographics and operator experience for each group were well matched between VL and DL groups; these are detailed in Table 3.

**Table 3:** Baseline demographics for patients randomised to direct laryngoscopy (DL) or videolaryngoscopy (VL) group. Values are median (IQR) or number (%)

	Direct laryngoscopy (*n* = 49)		Video laryngoscopy (*n* = 51)	
Age in years: median (Q1-Q3)	52 (41–64)		54 (40–67)	
Gender: number of male (%)	30 (61)		25 (49)	
BMI: median (Q1-Q3)	27.8 (24.7–32.4)		28.8 (25.0–31.8)	
ASA: number (%)				
1	8 (16.3)		9 (17.6)	
2	28 (57.1)		29 (56.9)	
3	13 (26.5)		13 (25.5)	
Surgical Acuity: number (%)				
Elective	36 (73.5)		35 (68.6)	
Scheduled > 24 h	5 (10.2)		8 (15.7)	
Urgent < 24 h	8 (16.3)		8 (15.7)	
Airway Difficulty Score (median, Q1-Q3)	7 (6–8)		7 (6–7.5)	
Comorbidities: number (%)				
Obesity	7 (14)		12 (24)	
OSA	3 (6)		3 (6)	
Surgical specialty: number (%)				
General	25 (51)		25 (49)	
Orthopaedic	4 (8)		8 (16)	
Urology	5 (10)		3 (6)	
Vascular	0 (0)		3 (6)	
Neurosurgery	11 (22)		8 (16)	
Device (number, %)	MAC	53 (100)	McGRATH	27 (53)
			GlideScope	20 (39)
			Unspecified	6 (8)
Operator grade and experience: number of operators (average years)				
Consultant	22 (15.2)		24 (12.5)	
Fellow	5 (5)		5 (5)	
Registrar	20 (3.5)		21 (3.9)	
SHO	1 (1)		0 (0)	

Feasibility outcomes are detailed in Table 4. Patient recruitment of 100 patients took 16 months, not achieving the 12-month recruitment target. Group adherence and data completeness were 100% and 97%, respectively. The factors leading to delaying the recruitment target could be avoided in a larger trial, as there would be adequate resource allocation to facilitate this.

**Table 4:** Feasibility outcome results for pilot trial. Values are in percentage

	Feasibility targets	Results
Recruitment rate (months to recruit 100 patients)	12 months	16 months
Data completeness	95%	97%
Adherence to group allocation	95%	100%
Proportion first-pass intubation success	≤ 85%	78%

The primary outcome of first-pass intubation success rate was 78% overall; with a rate of 83.7% (95% CI 70.3–92.7) in the DL group and 72.5% (95% CI 58.3–84.1) in the VL group (Table 5).

**Table 5:** Primary and secondary outcome results for patients intubated with direct laryngoscopy (DL) or videolaryngoscopy (VL). Values are median (Q1-Q3) or % and 95% confidence interval (95% CI)

	Direct laryngoscopy (*n* = 49)	Video laryngoscopy (*n* = 51)	*p* value
Primary outcome			
First-pass success: % (95% CI)	83.7 (95% CI 70.3–92.7)	72.5 (95% CI 58.3–84.1)	0.18*
Secondary outcome			
Time to intubate: seconds median (Q1-Q3), (95% CI)	34 (25–46), (95% CI 29–39)	43 (29–65), (95% CI 35–52)	0.038°
Intubation Difficulty Score: median (Q1-Q3), (95% CI)	1 (0–2), (95% CI 0–1)	0 (0–1.5), (95% CI 0–1)	0.13°
Complication rate^‡^: % (95% CI)	6.1 (95% CI 1.3–16.9)	7.8 (95% CI 2.2–18.9)	0.74*

*Pearson’s Chi-squared test

°Wilcoxon Rank-sum (Mann-Whitney) test

^‡^Complications include mucosal trauma, airway bleeding

Time to intubate was significantly shorter in the DL group when compared to the VL group (*p* = 0.038). Time to intubate and IDS were reported as median (Q1-Q3) as the distribution of these variables were non-normal. Median intubation time was 34 (95% CI 29–39) seconds in the DL group and 43 (95% CI 35–52) seconds in the VL group. The median IDS score for DL was 1 (95% CI 0–1) compared with 0 (95% CI 0–1) for the VL group; however, the difference was not statistically significant (*p* = 0.14) (Table 5). Complication rates for DL and VL were 6.1% (95% CI 1.3–16.9) and 5.9% (95% CI 2.2–18.9), respectively (*p* = 0.74); these included mucosal trauma and airway bleeding which are recognised complications of endotracheal intubation.

## Discussion

The aim of this study was to determine feasibility for a larger definitive trial. A first-pass intubation success rate of less than 85% would be required to produce a clinically viable, adequately powered study. This is an expected rate of first-pass success, as derived from other studies [4], and allows for a meaningful difference to be identified. Small differences in first-pass success rate may result in clinically important differences in overall intubation success after repeated attempts, avoid repeated airway trauma and reduce incidence of failed intubation. Our study revealed an overall first-pass success rate of 78%. This pilot study was not designed to be adequately powered to detect a clinically significant difference in first-pass intubation success between techniques. However, we have shown that a larger trial assessing a robust endpoint of first-pass success is feasible.

A recent meta-analysis performed by Lewis et al. [4] found no significant difference in first-pass success rate between VL and DL, with an overall first-pass intubation rate of 84%. Authors commented there was a moderate level of bias in this outcome measure due to heterogeneity of the included trials. They concluded that further research was likely to have an important impact on the estimate [4]. First-pass success rate was chosen as a primary endpoint as it is associated with less adverse incidents and greater intubation proficiency [10, 20]. Additionally, use of laryngoscopic view as a primary endpoint when comparing video and direct laryngoscopy does not necessarily equate to improved success rates of tracheal intubation [4]. There is insufficient data on first-pass success rates of DL and VL, which could be answered with a trial with sufficient sample size.

The use of videolaryngoscopes in clinical practice is now widespread and they have been shown to be a useful tool in achieving intubation in particular patient groups [21]. Much of the evidence comparing VL to DL and the conclusions made have been derived from heterogeneous trials of low participant numbers [4], ranging from less than 50 [22–26] to less than 150 participants [27–33]. Trials with more than 200 participants were limited to 1 to 5 operators [6, 34, 35] or did not report operator experience or proficiency in use of VL [36, 37]. This potentially limits the ability to interpret the results when applying them to a larger clinical environment. Our study is a pragmatic trial, where clinicians with a wide range of expertise and experience were utilised, with no discernible difference in the operator experience between the two groups. It is also a pilot trial, where relevant harms and benefits of each technique are measured to give overall data on potential harms. A larger trial will include a number of operators with a range of experience and a wide range of patient groups in order to test effectiveness of VL and DL and have adequate sample size to test for a clinically meaningful difference in the proportion of first-pass intubation success. This design would enable results to be more generalisable to real world situations.

Time to successful intubation has previously been examined in multiple different studies [21, 38, 39]; however, due to significant heterogeneity, no effective estimates have been drawn for this outcome in recent meta-analyses [4]. Our pilot study showed a significantly longer time to successful intubation using VL. The clinical significance of this finding will likely become more relevant in patient populations with a high metabolic requirement, or high oxygen consumption, where desaturation occurs more rapidly, e.g. obesity, pregnancy and emergency surgery. Many reasons for a difference in time to intubation success have been speculated. By providing a direct line from the patient’s mouth to the larynx, passage of the endotracheal tube during DL is intuitive and often does not require the use of a pre-formed stylet. During VL, however, particularly those with a hyper-angulated blade, a different technique is required to manipulate a pre-formed stylet and endotracheal tube through the vocal cords. This difference in intubation technique may account for the difference in time taken to achieve successful intubation.

There is a learning curve for both DL and VL before technical proficiency is achieved. There is no agreed definition on what constitutes competency in using videolaryngoscopes. Some authors advocate greater than 20 intubations, [4, 40, 6] while others suggest 76 intubations are required before competency is achieved [41]. We used a minimum of 20 intubations as our operator exclusion criteria. In deciding the benchmark for operator competency, we took a pragmatic approach. Operators who had used a VL at least 20 times in clinical practice would consider themselves independent, whereas a benchmark of 76 could have resulted in a narrow range of expert operators. We acknowledge the use of 20 intubations with the chosen device may introduce operator bias; however, we believe there needs to be a range of operator experience to appropriately test effectiveness. The degree of operator experience and potential bias will be re-evaluated prior to undertaking a larger clinical trial. Examining operator experience will also provide additional evidence on the effect of experience on the efficacy of VL, an area of research which is currently lacking [4].

This trial was designed to test technique of indirect video laryngoscopy rather than comparison of specific devices. The videolaryngoscope technique implemented used a rigid laryngoscope to displace soft tissues and transmit a picture from the tip of the laryngoscope to a monitor, thus enabling an indirect view of the glottis. The models of videolaryngoscope used in the trial were the GlideScope® AVL System (Verathon, Washington) and McGRATH-MAC™ (Medtronic, Minneapolis), which can both be used with the technique described. However, proficiency in one video-assisted device does not equate to proficiency with all types of videolaryngoscopes [42]. We allowed the procedural anaesthetist to choose the type of device according to their preference. All anaesthetists had at least 1 year of clinical experience. The trial is designed to assess the first-pass intubation success rates within a tertiary institution, using the devices available to them within their working environment, and therefore, it is possible that some practitioners had not reached the plateau of the learning curve for each device. Operator experience and VL learning curves are both areas of potential bias for testing the effectiveness of indirect VL and would provide valuable information on the implications for use of VL. This is beyond the scope of a feasibility trial but would be further evaluated when designing a larger randomised controlled trial.

During the trial, we reflected that our protocol could have allowed deviation from a solely indirect VL technique. We acknowledge that an improvement in a future trial would be a modification of the protocol to limit the videolaryngoscope technique to indirect VL only, to ensure that this technique was being tested. Although two devices were used to test the effectiveness of indirect VL in this pilot, we have demonstrated that comparison of indirect VL technique with DL in a larger randomised controlled trial is feasible.

There are a number of limitations with the trial design. Firstly, it is impossible to blind the operator from the technique being used and this could be a potential source of operator bias. Although outcome assessors were also unblinded to the technique, the primary and secondary outcomes were objective measures, limiting the relevance of blinding at this level. Secondly, in our institution, neuromuscular monitoring to assess degree of motor blockade is not routinely used prior to intubation. We acknowledge this limitation, however the intent of this feasibility trial was to test regular clinical practice, where assessing neuromuscular blockade prior to intubation is not routine. This practice was also consistent between the two cohorts of patients in the trial. Thirdly, the IDS used to compare DL and VL may not be the optimal comparison tool to assess differences in intubating ability. The IDS has not been validated for use in VL and has demonstrated poor correlation between the score and user-rated intubation difficulty in VL [43]. Unfortunately, at present, there is no validated scoring system designed to assess intubation difficulty using indirect videolaryngoscopes. We are not aware of any validated scoring systems specifically designed to compare VL with DL. Fourthly, we did not specify on the CRF when complication details were to be recorded. Within our study, complication rates were primarily recorded immediately following intubation; however, we acknowledge that evidence of laryngeal trauma may only become evident following extubation.

As this was a pilot study where the primary aims are feasibility aims, a non-inferiority trial design was not considered. In the subsequent large trial we are designing, a non-inferiority design is one potential design. However, the primary scientific results from our pilot are somewhat counterintuitive in that traditional laryngoscopy appears to be superior. Our pilot results are evidence not in favour of a non-inferiority design for a subsequent large trial, as there appears to be equipoise as to the superiority of VL or DL, but favour a superiority trial design.

## Conclusions

In summary, this pilot study shows that it is feasible to perform a large randomised controlled trial with first-pass intubation as the primary endpoint. Plans are underway to perform an adequately powered randomised controlled trial comparing the effectiveness of a specific VL technique to DL and to assess operator experience and complications in greater detail.

## Abbreviations

ADS: Airway Difficulty Score
CRF: Case report form
DL: Direct laryngoscopy
IDS: Intubation Difficulty Score
OELM: Optimal external laryngeal manipulation
PACU: Post Anaesthetic Care Unit
VL: Videolaryngoscopy
